# Identification of highly penetrant Rb-related synthetic lethal interactions in triple negative breast cancer

**DOI:** 10.1038/s41388-018-0368-z

**Published:** 2018-06-18

**Authors:** Rachel Brough, Aditi Gulati, Syed Haider, Rahul Kumar, James Campbell, Erik Knudsen, Stephen J. Pettitt, Colm J. Ryan, Christopher J. Lord

**Affiliations:** 1 The Breast Cancer Now Toby Robins Breast Cancer Research Centre, London, SW3 6JB UK; 2 CRUK Gene Function Laboratory, The Institute of Cancer Research, London, SW3 6JB UK; 3 Department of Medicine, University of Arizona, Tucson, AZ 85721 USA; 4 Systems Biology Ireland, University College Dublin, Dublin, Ireland; 5 School of Computer Science, University College Dublin, Dublin, Ireland

## Abstract

Although defects in the *RB1* tumour suppressor are one of the more common driver alterations found in triple-negative breast cancer (TNBC), therapeutic approaches that exploit this have not been identified. By integrating molecular profiling data with data from multiple genetic perturbation screens, we identified candidate synthetic lethal (SL) interactions associated with *RB1* defects in TNBC. We refined this analysis by identifying the highly penetrant effects, reasoning that these would be more robust in the face of molecular heterogeneity and would represent more promising therapeutic targets. A significant proportion of the highly penetrant *RB1* SL effects involved proteins closely associated with RB1 function, suggesting that this might be a defining characteristic. These included nuclear pore complex components associated with the MAD2 spindle checkpoint protein, the kinase and bromodomain containing transcription factor TAF1, and multiple components of the SCF^SKP^ Cullin F box containing complex. Small-molecule inhibition of SCF^SKP^ elicited an increase in p27^Kip^ levels, providing a mechanistic rationale for RB1 SL. Transcript expression of SKP2, a SCF^SKP^ component, was elevated in *RB1*-defective TNBCs, suggesting that in these tumours, SKP2 activity might buffer the effects of *RB1* dysfunction.

## Introduction

Patients who develop triple-negative breast cancer (TNBC), i.e., those breast cancers that lack amplification of the *ERBB2* gene as well as expression of both the oestrogen and progesterone receptors, tend to have a relatively poor prognosis and represent a significant area of unmet clinical need, where novel therapeutic approaches are acutely needed (recently reviewed in ref. [1]). Although some targeted approaches have been proposed for molecularly defined subsets of TNBC patients, for most, classical chemotherapy regimens still represent the mainstay of treatment, making the requirement to identify novel targets in this disease critical. One approach to this problem has been to define the molecular composition of TNBCs and then to use this information to help identify therapeutic vulnerabilities that might operate in the disease. Already, the delineation of genomic, transcriptomic and proteomic profiles of tumours has identified a series of distinct molecular subtypes of TNBC, as well as identifying likely cancer driver gene mutations that operate in the disease [2].

One of the more frequent driver alterations in TNBCs involves deleterious mutations (e.g., truncating mutation, gene deletions, etc.) in the *Retinoblastoma* tumour suppressor gene (*RB1* aka *Rb*). In non-tumour cells, Rb’s canonical role is in cell cycle progression, a function mediated in part by the repressive effect Rb has on the E2F family of transcription factors [3]. A somewhat reductionist model of Rb’s role in tumour suppression suggests that loss of Rb’s E2F repressive function allows premature transition of cells through the G_1_ cell cycle checkpoint; it also seems likely that loss of Rb function in breast cancer also influences additional processes that contribute to the development of the disease, including the differentiation of stem and progenitor cells and the transition of cells from an epithelial to a mesenchymal phenotype [3].

Commensurate with its key role in cell cycle control, genomic alterations in the *RB1* gene are relatively common in TNBCs [4–6] as well as in a series of other malignancies [7–9]. In TNBC, loss of Rb protein expression is found in >40 % of cases [10, 11] (and reviewed in ref. [12]). Although patients with Rb-defective tumours (as defined by immunohistochemistry and/or gene expression) tend to have a relatively favourable response to classical chemotherapy [13–15] many either fail to respond to therapy or later relapse with therapy-resistant disease, suggesting that novel therapeutic approaches are required to target this patient subset.

One approach to identifying novel therapeutic targets that could be exploited in patients with specific tumour suppressor gene defects has been to identify synthetic lethal interactions associated with these genes. For example, the identification of synthetic lethal interactions between *BRCA1* or *BRCA2* tumour suppressor genes and inhibition of the PARP1 DNA repair protein has driven the clinical development and approval for use of PARP inhibitors for the treatment of cancer [16]. One notable feature of the BRCA/PARP1 synthetic lethal effect, which contributes to its translational value, is that it is highly penetrant [17]; i.e., in otherwise molecularly diverse pre-clinical models, and cancer patients, the presence of the predictive biomarker, in this case *BRCA1* or *BRCA2* mutation, more often than not predicts a profound antitumour cell response to a PARP inhibitor. This highly penetrant nature suggests that this particular synthetic lethal effect is robust in the face of the molecular heterogeneity that exists between different human cancers. Here we describe efforts to identify highly penetrant synthetic lethal effects associated with deleterious Rb alterations in TNBC; we reasoned that those with greatest penetrance will be more likely to operate in the diverse molecular contexts within the TNBC subtype and thus represent more promising therapeutic targets. Although genes such as *TSC2* [18] and elements of the Dicer pathway [19] have been shown to be synthetic lethal with Rb defects, as far as we are aware, the penetrance of these effects, or whether these operate in models of TNBC, has not as yet been assessed. The availability of several, large-scale, short hairpin RNA (shRNA) and small interfering RNA (siRNA) screens [20–23], conducted in multiple tumour cell lines (TCLs), some of which are derived from TNBCs, now make a detailed identification of highly penetrant RB1-related synthetic lethal effects now possible. For this reason, we describe here a detailed molecular analysis of Rb status in TCLs derived from TNBC. We then designed a straightforward data analysis pipeline that allowed us to use this Rb annotation to interrogate both in-house and publically available large-scale, shRNA and siRNA screens to identify candidate Rb-related synthetic lethal effects. Within this data analysis pipeline, we included an estimate of penetrance. In triaging the candidate Rb-synthetic lethal effects that operated in TNBC tumour cells to identify those with greatest penetrance, we identified a series of pharmacologically tractable effects, one of which, SKP2, we validated using both genetic and pharmacological methods. We also noted that a significant proportion of the highly penetrant Rb SL effects in TNBC involved proteins closely associated with Rb function, suggesting that this might be a defining characteristic.

## Results

### Annotation of Rb status in TNBC TCLs

**Fig. 1:**
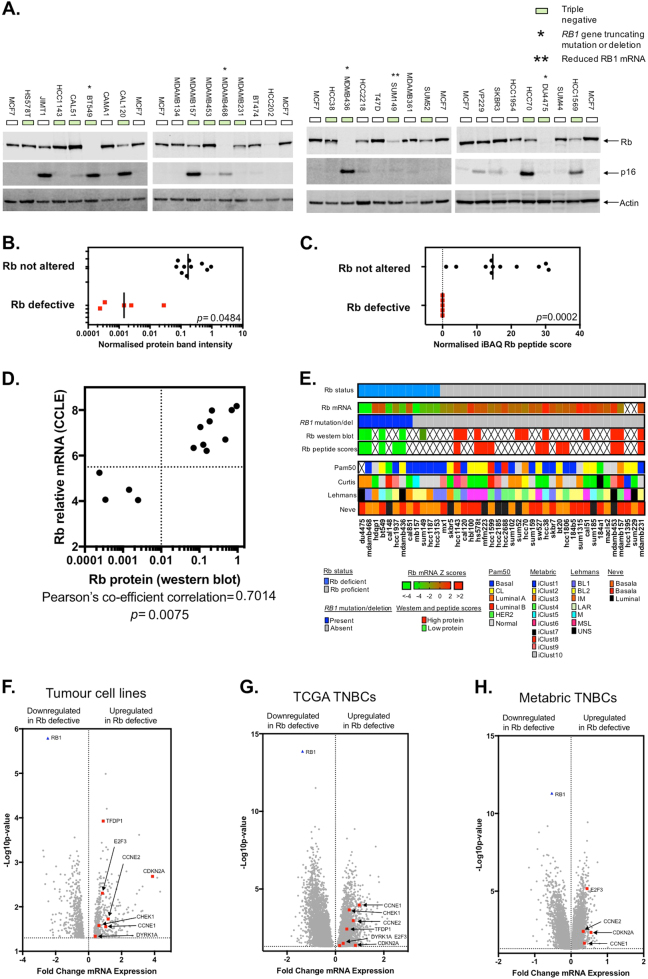
Rb status in TNBC tumour cell lines. **a** Western blot illustrating Rb and p16 protein expression in 30 breast tumour cell lines (TCLs). BT549, MDAMB436, MDAMB468 and DU4475 each possess loss-of-function mutations in the *RB1* gene. SUM149 cells express reduced *RB1* mRNA. **b** Scatter plot illustrating quantification of Rb protein levels delineated from **a**. Protein expression in Rb-defective vs. not altered, *p* = 0.0484, Student’s *t* test. **c** Scatter plot illustrating Rb protein expression in defective and proficient TNBC TCLs estimated by mass spectrometry data from ref. [24]. TNBC TCLs were classified into “Rb-defective” and “not altered” groups according to western blot data from **a**; using this classification, normalised iBAQ Rb peptide scores were compared and are shown. *p* = 0.0002, Fishers exact test. **d** Scatter plot illustrating the correlation between Rb protein and mRNA transcript levels in TNBC TCLs. Rb protein levels from **a** and **b** are compared to Rb mRNA transcript levels obtained from the CCLE database [25]. Correlation *r* = 0.7, *p* = 0.0075, Pearson’s correlation. **e** Oncoprint illustrating Rb and breast cancer subtype status amongst 42 TNBC TCLs. **f** Volcano plot illustrating mRNAs that are differentially expressed (limma analysis *p* < 0.05) in Rb-defective (vs. Rb not altered) TNBC TCLs. Genes functionally related to Rb are highlighted, as is Rb itself. **g** Volcano plot of mRNAs that are differentially expressed (limma analysis *p* < 0.05) in 48 Rb-defective (vs. Rb not altered) triple-negative breast tumours from the TCGA study [27]. Genes functionally related to Rb are highlighted, as is Rb itself. **h**.Volcano plot of mRNAs that are differentially expressed (limma analysis *p* < 0.05) in 55 Rb-defective (vs. Rb not altered) triple-negative breast tumours from the Metabric study [28]. Genes functionally related to Rb are highlighted, as is Rb itself

To identify highly robust synthetic lethal effects associated with Rb defects in TNBC, we classified a molecularly diverse panel of TNBC TCLs according to Rb status and then used this Rb classification to interrogate publically available genetic screen data using a data analysis pipeline (described later) that identified highly penetrant synthetic lethal effects. To do this, we first classified TNBC TCLs using a combination of genomic, transcriptomic and proteomic data to identify those with Rb genetic or epigenetic defects. We used western blotting to assess Rb protein expression in 30 breast TCLs, including 16 TNBCs (Fig. 1a, b). In this analysis, we found that TCLs with deleterious mutations in the *RB1* gene (BT549, *RB1* c.265_607del343; MDAMB468, *RB1* c.265_2787del2523; DU4475, *RB1* c.1_2787del2787; and MDAMB436, *RB1* c.607_608ins107) lacked Rb protein expression, suggesting that the mutational status of *RB1* correlated with protein expression (Fig. 1a, b). We also found that SUM149 cells exhibited low Rb protein expression, an observation we found to be consistent with reduced *RB1* mRNA levels (Supplementary Data 1). To assess Rb protein status by orthogonal means, we compared our western blot data with publically available mass spectrometry data describing the proteomic profiles of 16 TNBC TCLs [24]. Using average intensity-based absolute protein abundance (iBAQ) data for Rb from mass spectrometry profiling [24] (Supplementary Data 1), we found that TNBC TCLs classified by western blotting as being Rb-defective exhibited no Rb peptides (MDAMB468, MDAMB436, HCC1937, DU4475, BT549) when compared to those TCLs we had classified as Rb-proficient (Fig. 1c, p = 0.0002), giving us some confidence in our classification. Examination of transcriptomic profiles of TNBC TCLs [25] revealed that TCLs with reduced levels of *RB1* mRNA exhibited low Rb protein expression (Fig. 1d, *p* = 0.0075), suggesting that *RB1* mRNA expression levels could be used as a reasonable proxy for protein expression. Taking proteomic, genomic and transcriptomic data into consideration (Fig. 1e), we then classified a total of 42 TNBC TCLs according to Rb status, identifying 12 with one or more defects in Rb (“Rb-defective”, e.g., low protein expression, truncating mutation/gene deletion and reduced mRNA levels: BT549, HCC1937, DU4475, MDAMB436, MDAMB468, CAL148, HDQP1, MB157, SUM149, HCC1187, HCC3153 and CAL851) and 30 TNBC TCLs as not exhibiting such defects (“Rb not altered” TCLs).

To further assess the validity of our Rb classification, we assessed the transcriptomic profiles of Rb-defective TNBC TCLs to assess whether these reflected loss of Rb function (Fig. 1f). Using the Rb-defective and Rb not altered classification described above for 42 TNBC TCLs, we identified 839 differentially expressed genes (452 with reduced expression in Rb-defective TCLs, 387 with elevated expression (*p* < 0.05, limma analysis, Supplementary Data 2)). As expected, we found *RB1* itself to be the fifth most downregulated gene in the Rb-defective TCLs compared to Rb not altered TCLs (log fold change of −2.4, *p* value = 1.6 × 10^−6^ (limma analysis); Supplementary Data 2 and Fig. 1f). We also found that Rb-defective TCLs exhibited elevated expression of cyclins associated with G_1_ checkpoint control and S phase progression (cyclin E1 (*p* = 0.03), E2 (*p* = 0.02) and CDKN2A (p16; *p* = 0.002)), as well as elevated expression of the Rb-regulated E2F3 transcription factor and its binding partner TFDP1 (Fig. 1f (*p* = 0.005 and 0.0001, respectively)). Using the ENRICHR annotation tool [26] to identify pathways rather than individual genes that were differentially expressed in the Rb-defective group, we found that pathways related to Rb and G_1_ to S cell cycle control to be among the most significantly dysregulated in Rb-defective TNBC TCLs, including “Retinoblastoma (RB) in Cancer WP2446” *p* = 1.7 × 10^−11^ and “G_1_ to S cell cycle control WP45” *p* = 1.4 × 10^−9^, both of which included genes such as *MCM4*, *6* and *7*, *TFDP1*, *CCNE1*, *CCNE2*, *CHEK1*, *E2F3* and *RBP1*. Using the same annotation tool we also searched for the key transcription factors that regulated genes that were differentially expressed in Rb-defective TNBC TCLs. We found that a significant proportion of the differentially expressed genes in Rb-defective TNBC TCLs were targets of Rb-related E2F-family transcription factors, including E2F7, E2F4 and E2F1 (e.g., *p* = 0.001, 0.005 and 9.3 × 10^−7^ for E2F7, E2F4 and E2F1, respectively).

To compare the observations made in TCLs with TNBC tumours, we used the same approach of exploiting *RB1* gene mutation/copy number status and RB1 mRNA expression profiles to classify 140 The Cancer Genome Atlas (TCGA) triple-negative breast tumours [27] according to Rb status; this approach identified 48 Rb-defective TNBC tumours and 92 where Rb was not altered. Assessing the transcriptomic profiles of these TNBCs we again found that genes associated with Rb and Rb-related G_1_ to S cell cycle control were frequently dysregulated in Rb-defective TNBCs, including *CDKN2A*, *TFDP1*, *CCNE1*, *CCNE2*, *E2F3*, *CHEK1* and *DYRK1A*, a recently identified *RB1* synthetic lethal gene [21] (Fig. 1g, Supplementary Data 3), consistent with the observations made in TNBC TCLs. We also applied the same approach to classify 182 Metabric TNBC tumours [28] according to Rb status; this approach identified 55 Rb-defective and 132 Rb not altered TNBC samples. Assessing the transcriptomic profiles of these TNBCs we again found that genes associated with Rb and Rb-related G_1_ to S cell cycle control were frequently dysregulated in Rb-defective TNBCs (Fig. 1h, Supplementary Data 4). These global transcriptional patterns in Rb-defective TNBC TCLs and human tumours suggested that our Rb classification approach was somewhat valid.

### A pipeline for the identification of highly penetrant Rb synthetic lethal effects in TNBC

**Fig. 2:**
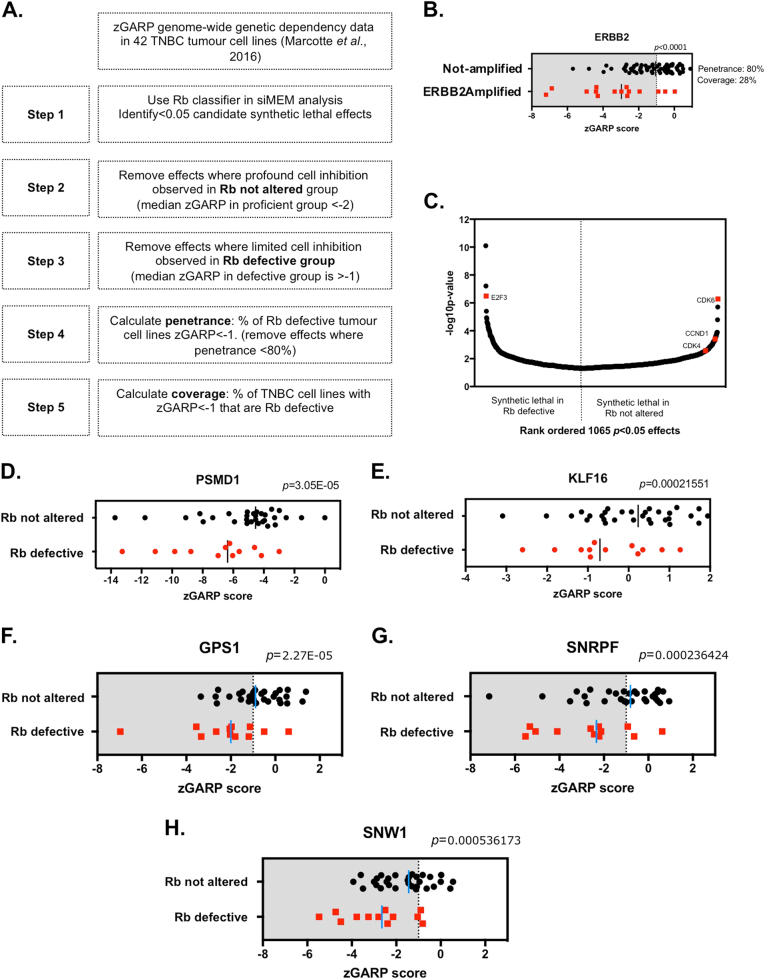
Identifying highly penetrant Rb synthetic lethal effects that operate in TNBC. **a** Schematic illustrating the data analysis workflow used. In the first instance, 16 056 gene zGARP scores from shRNA screens in 42 TNBC cell lines described in the Colt2 data set were analysed; parallel analyses were carried out using data from the DRIVE and Achilles data sets (see main text). **b** Scatter plot illustrating *ERBB2* zGARP scores in 77 breast tumour cell lines partitioned according to effects in ERBB2-amplified and -non-amplified TCLs. *ERBB2* shRNA elicits a *p* < 0.0001 oncogene addiction effect (siMEM) with 80% penetrance in *ERBB2*-amplified tumour cell lines (red). Coverage is also shown. **c** Scatter plot illustrating 1065 *p* < 0.05 significant siMEM Rb synthetic lethal effects identified from the siMEM analysis of TNBC TCLs in the *Colt2* study (step one in **a**). *p* < 0.05 effects are ranked ordered by siMEM *p* value. E2F3 (synthetic lethal in Rb-defective), CDK6, CDK4 and the CDK4,6 cyclin partner, Cyclin D1 (*CCND1*) (dependencies in Rb not altered) are highlighted. **d**, **e**. Scatter plots illustrating *Z* scores in 42 TNBC TCLs for two siMEM *p* < 0.05 candidate Rb synthetic lethal effects, PSMD1 (D) and KLF16 (E), removed from further analysis by the use of *Z* score filters (steps two and three in **a**). zGARP scores for *PSMD1* (preferentially target Rb-defective, siMEM *p* value 3 × 10^−5^) indicate all but two Rb not altered tumour cell lines exhibit *Z* score of <−2 (median *Z* in not altered group < −4). zGARP scores for *KLF16* (preferentially target Rb-defective, siMEM *p* value 0.0002) indicate that the majority of Rb-defective TCLs exhibit *Z* score of >−1 (median *Z* in defective group = −0.8), despite median *Z* scores being significantly different in Rb not altered vs. deficient TCLs. **f**–**h** Scatter plots illustrating *Z* scores in 42 TNBC TCLs for three siMEM *p* < 0.05 candidate Rb synthetic lethal effects, GPS1, SNRPF and SNW1, where median *Z* in defective group < −1 and median *Z* in proficient group > −2 (steps two and three in **a**)

Using the Rb classification of TNBC TCLs described above, we re-analysed publically available genetic screen data (e.g., genome-wide shRNA screen data [22, 23], gene subset shRNA screen data [29] or gene subset siRNA data [20, 21]) from TNBC TCLs, using a series of iterative data-processing steps designed to identify highly penetrant synthetic lethal effects (Fig. 2a). In summary, these steps involved: (i) step one—identification of candidate synthetic lethal effects: using shRNA screen data [22] for 12 Rb-defective TNBC cell lines and 30 Rb not altered cell lines we used the si/shRNA mixed effect model (siMEM) algorithm [22] to identify those genes whose inhibition appeared to target the Rb-defective models to a greater extent than Rb-proficient TNBC TCLs (*p* < 0.05). (ii) Steps two and three—apply *Z* score thresholds to identify profound cell inhibitory effects: although step one allowed us to identify genes whose inhibition selectively targeted Rb-defective TCLs to a greater extent than Rb not altered TCLs, we reasoned that the scale of cell inhibition in these two TCL cohorts might also be critical in identifying the most suitable therapeutic targets. For example, for the purposes of identifying novel therapeutic targets we were less interested in genes whose inhibition profoundly inhibited both Rb-defective and Rb not altered groups, even if the Rb-defective models exhibited significantly greater sensitivity; we assumed that inhibition of these targets would likely cause significant normal cell toxicity. Similarly, we also assumed that candidate synthetic lethal effects that did not elicit profound cell inhibitory effects in Rb-defective TCLs would be less likely to elicit profound anticancer therapeutic effects when exploited clinically. For these reasons, we triaged the list of candidate synthetic lethal effects identified in step one to remove from further analysis: (a) those genes that appeared to be synthetic lethal with Rb but whose targeting elicited profound cell inhibition in Rb not altered TCLs; and (b) those genes that appeared to be synthetic lethal with Rb but whose targeting did not elicit profound cell inhibitory effects in Rb-defective TCLs. To do this we used shRNA/siRNA *Z* score thresholds to estimate the effect of each RNAi reagent. (iii) Steps four and five—identify effects with greatest penetrance: we reasoned that the most clinically effective synthetic lethal targets are likely to be those that have complete or highly penetrant effects, i.e., the presence of the predictive biomarker (in this case an Rb defect) is more often than not associated with profound sensitivity to target inhibition. For this reason, we finally triaged synthetic lethal effects by calculating synthetic lethal penetrance (SLP) scores; in this case, SLP for each synthetic lethal effect was calculated as the percentage of Rb-defective TCLs that exhibited a *Z* score of <−1 when targeted with shRNA. We also calculated synthetic lethal coverage (SLC) scores, i.e., the percentage of TNBC TCLs that were sensitive to shRNA that were Rb-defective, to estimate the specificity of synthetic lethal effects for Rb defects.

To illustrate the concepts of penetrance and coverage as applied to functional genomic screens in TCLs, we examined a well-characterised and therapeutically actionable oncogene addiction effect that operates in breast cancer, namely that associated with amplification of the epidermal growth factor receptor oncogene, *ERBB2*. Amplification and overexpression of *ERBB2* is used clinically to stratify breast cancer patients for treatment with ERBB2-targeting agents such as the monoclonal antibody trastuzumab [30]. In examining the Colt2/Marcotte et al. shRNA data set (78 432 shRNAs targeting 16 056 genes in 77 breast TCLs representing TNBC and non-TNBC subtypes [22]), we found that as shown before [22], that shRNA targeting of *ERBB2* selectively targeted *ERBB2*-amplified breast TCLs (siMEM, *p* < 0.0001), elicited minimal inhibition in *ERBB2* non-amplified TCLs (median *Z* −1), profound inhibition in *ERBB2*-amplified TCLs (median Z -3) and had a SLP (penetrance) score of 80% and a SLC (coverage) score of 28% (Fig. 2b); the high penetrance score in this case, taken together with ERBB2 fulfilling the other selection criteria, reconfirms *ERBB2* as a suitable therapeutic target in appropriately stratified breast cancer patients.

### Identification of Rb synthetic lethal effects from shRNA screens

To identify Rb synthetic lethal effects, we first used genome-wide shRNA data from 42 TNBC TCLs described in the Colt2/Marcotte et al. study [22]. In this data set, the combined effects of multiple shRNAs targeting a single gene are described as *Z* normalised Gene Activity Ranking Profile (zGARP) scores [31]. We re-analysed this shRNA screen data using the siMEM mixed effect linear model method (see step one, above), which identifies synthetic lethal effects by estimating the depletion or “dropout” rate of individual shRNAs in cohorts of cell lines classified according to a molecular feature [22], in this case Rb deficiency. Using the siMEM approach with our Rb classification of 42 TNBC TCLs, we identified 1065 Rb-specific dependencies (*p* < 0.05, siMEM, Fig. 2c): 437 genes, where shRNA preferentially inhibited Rb-defective TNBC (i.e. Rb synthetic lethal effects) and 628 genes, where shRNAs preferentially targeted Rb-proficient TNBC TCLs (Supplementary Data 5). Amongst these, we noted that shRNAs targeting *CDK4*, *CDK6* or the CDK4,6 cyclin partner gene *Cyclin D1* (*CCND1*) preferentially inhibited Rb-proficient TNBC TCLs (Fig. 2c), consistent with the hypothesis that inhibition of CDK4,6 activity restores cell cycle control in Rb-proficient TNBC tumour cells and elicits cell inhibition [32]. In terms of identifying Rb dependencies, we noted that the siMEM analysis of the Colt2 data set identified shRNA targeting the E2F family transcription factor, E2F3, as being one of the most significant Rb synthetic lethal effects (Fig. 2c). These Rb-related observations thus gave us some confidence in the approach. We also carried out similar analyses in siRNA/shRNA data sets that included TNBC TCLs, from other sources: the DRIVE data set [29]; the Achilles data set [23]; and the ICR-Intercell data set [20, 21], and provide the lists of Rb dependencies identified in these data sets in Supplementary Data 6, 7, 8, respectively.

### Application of *Z* score thresholds identifies profound cell inhibitory effects

As described above, we were interested in identifying Rb synthetic lethal effects that had profound effects in Rb-defective TNBCs but had minimal effects in cells without Rb defects. Although approaches such as siMEM are useful in identifying putative vulnerabilities, they often do not, when used in isolation, identify synthetic lethal effects with these favoured characteristics. For example, Fig. 2d, e illustrates a pair of *p* < 0.05 Rb dependencies identified by siMEM analysis (step one), which had either profound cell inhibitory effects in both Rb not altered as well as Rb-defective TNBC TCLs, e.g., *PSMD1* (Fig. 2d, siMEM *p* = 3 × 10^−5^, median *Z* in Rb-defective of −6, median *Z* in Rb not altered of −4) or relative paucity of profound cell inhibition effects in the Rb-defective TCL cohort, e.g., *KLF16* (Fig. 2e, siMEM *p* = 0.0002, median *Z* in Rb-defective of −0.8). To eliminate such effects from further study, we applied a pragmatic approach that removed from further assessment *p* < 0.05 synthetic lethal effects where the median zGARP score in the Rb-defective TCLs was >−1 (i.e., effects, where profound cell inhibition in Rb TCLs not observed) and those effects where median zGARP score in Rb-proficient effects was <−2 (i.e., dependencies that still elicited profound cell inhibition in Rb-proficient TCLs); three examples that fulfilled these criteria, *GPS1*, *SNRPF* and *SNW1*, are shown in Fig. 2f, g, h. This triage step identified 122 Rb synthetic lethal effects in the Colt2 data set that fulfilled these criteria (Supplementary Data 9). Similarly, triaged dependencies were identified in the DRIVE [29], Achilles [23] and ICR-Intercell data sets [20, 21] (Supplementary Data 10, 11 and 12, respectively).

### Highly penetrant Rb synthetic lethal effects in TNBC include TAF1, TAF1 target genes, nuclear pore complex proteins and the SCF^SKP^-COP9 signalosome complexes

**Fig. 3:**
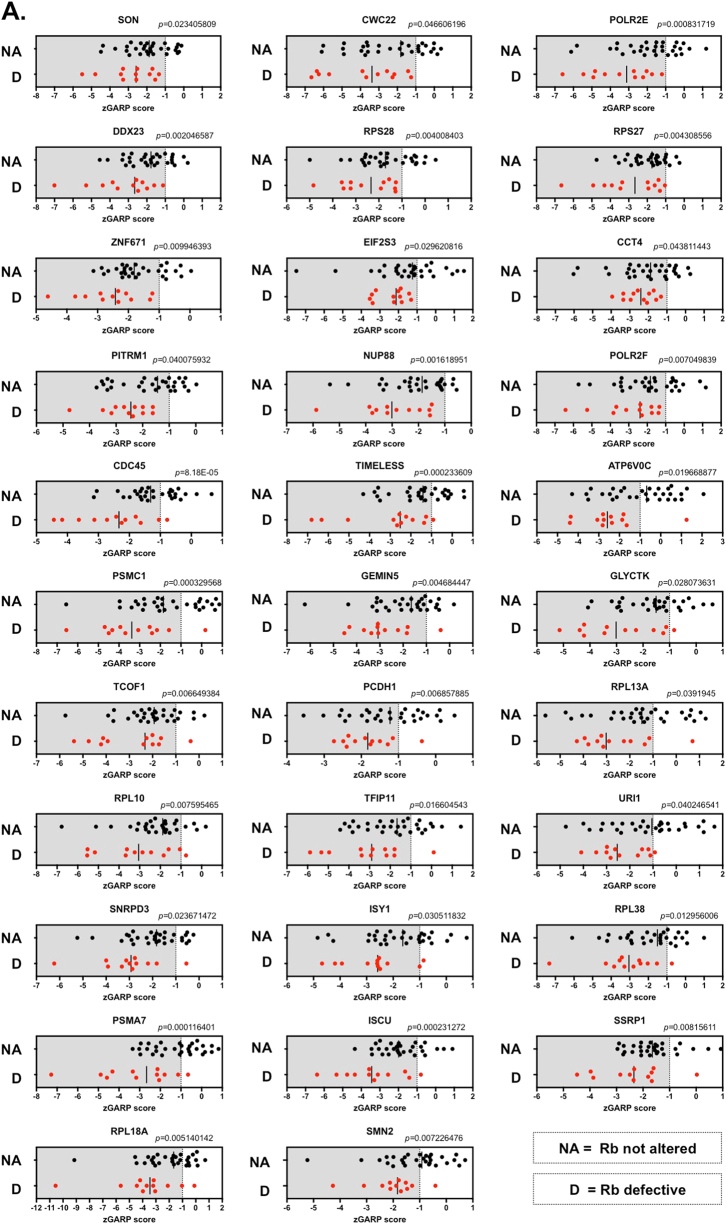
Highly penetrant Rb synthetic lethal effects. Scatter plots illustrating *Z* scores in 42 TNBC TCLs for 30 candidate Rb synthetic lethal effects, which pass *Z* score threshold assessment and demonstrate a penetrance score of >90%, as summarised in steps 1–5 of Fig. 2a

We next calculated SLP and SLC scores for each Rb synthetic lethal effect triaged in steps two and three. In our analysis of the *Colt2* data set, we calculated SLP and SLC scores for 122 Rb synthetic lethal effects, identifying 54 effects that had >80% penetrance (Fig. 3a (SLP > 90% shown), Supplementary Data 9) and also identified highly penetrant effects from our analysis of the DRIVE and Achilles and ICR-Intercell data sets (Supplementary Data 10, 11 and 12, respectively). Amongst the highly penetrant Rb SL candidates (>80% penetrant), a significant number is known to be involved in RNA splicing (Supplementary Figure 1, *p* = 5.14 × 10^−12^, GO Biological Processes 2017, ‘mRNA splicing, via spliceosome’, Enrichr [26], Supplementary Data 13), including: ISY1 (SLP = 92%); SON (SLP = 100%); CWC22 (SLP = 100%); DDX23 (SLP = 100%); POLR2E (SLP = 100%); GEMIN5 (SLP = 92%); POLR2F (SLP = 100%); TFIP11 (SLP = 92%); SRRM2 (SLP = 83%); LSM2 (SLP = 83%); SNW1 (SLP = 83%); SART3 (SLP = 83%); FIP1L1 (SLP = 83%); and SNRPD3 (SLP = 92). Interestingly, a RNAi screen in *Caenorhabditis elegans* also identified a synthetic lethal interaction between Rb loss and components of the splicing machinery [33]. Similarly, a number of proteins involved in the regulation of translation were also identified as candidate Rb SL targets (*p* = 1.7 × 10^−7^, GO Biological Processes 2017, ‘regulation of translation by machinery localisation’, Enrichr [26], Supplementary Figure 1, Supplementary Data 13). These included: RPS24 (SLP = 83%); RPS27 (SLP = 100%); RPS28 (SLP = 100%); RPL18A (SLP = 92%); RPL13A (SLP = 92%); RPL10 (SLP = 92%); TCOF1 (SLP = 92%); GEMIN5 (SLP = 92%); and RPL38 (SLP = 92%).

We also identified two nuclear pore complex (NPC) components [34, 35] NUP88 (SLP = 100%) and NUP214 (SLP = 83%) as highly penetrant Rb synthetic lethal partners (Supplementary Figure 1, Supplementary Data 13). NPCs are responsible for trafficking proteins between the nucleus and the cytoplasm. In particular, NPCs control spindle assembly, faithful chromosome segregation and mitotic progression by controlling the temporal and spatial localisation of proteins [36–38], including the E2F transcriptional target and spindle assembly checkpoint protein, MAD2, whose elevated expression is required for chromosomal instability in Rb-defective cells [39, 40]. It seems possible that the highly penetrant synthetic lethalities between Rb and NUP88 or NUP214 could be related to the temporal and spatial localisation of MAD2, perhaps by causing a level of excessive genomic instability that is incompatible with cell viability.

**Fig. 4:**
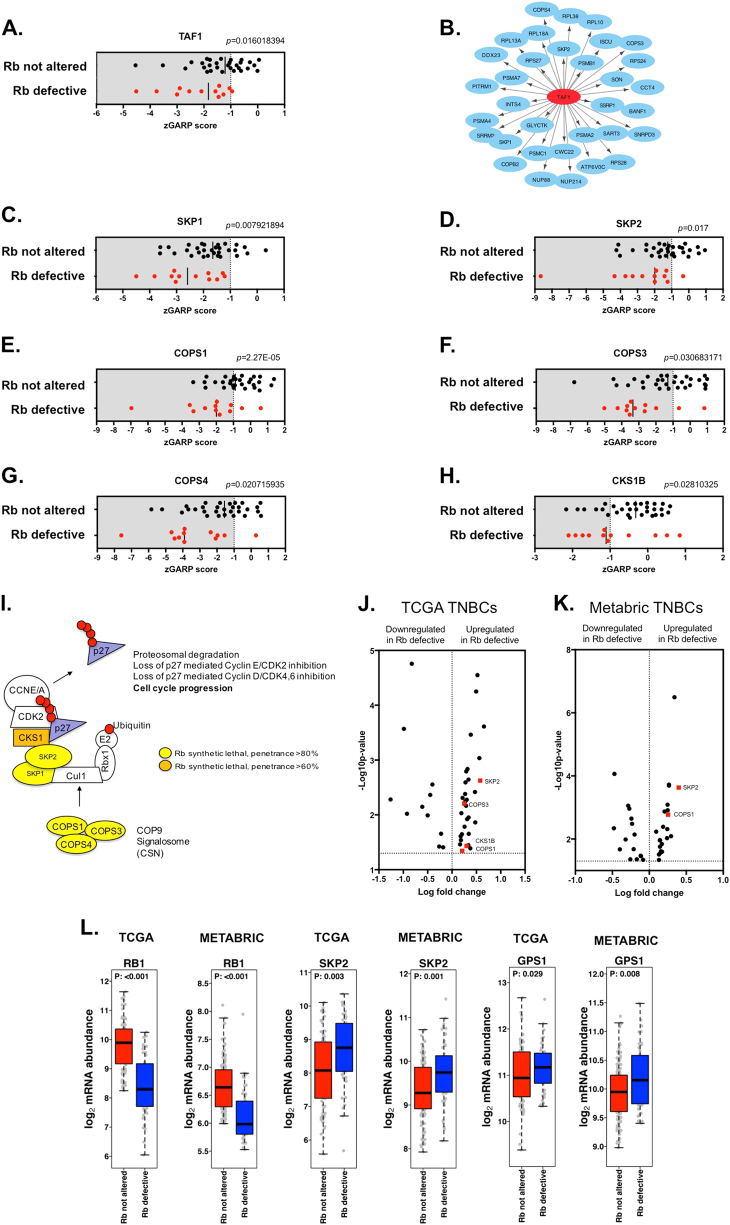
TAF1 and SKP2 as central nodes in highly penetrant Rb synthetic lethal networks. **a** Scatter plot illustrating *Z* scores in 42 TNBC TCLs for TAF1 from the data analysis illustrated in Fig. 2a. **b** Cytoscape network plot illustrating 33 highly penetrant (>80% penetrance) Rb synthetic lethal effects identified as TAF1 transcription factor target genes, as annotated by ENCODE data [26, 44]. **c**–**h** Scatter plots illustrating *Z* scores in 42 TNBC TCLs for SKP1, SKP2, COPS1,3,4 and CKS1B from the data analysis in Fig. 2a. **i** Pathway diagram highlighting the role of multiple highly penetrant Rb synthetic lethal effects in the control of p27 activity. **j** Volcano plot illustrating mRNAs from highly penetrant Rb SL genes that are differentially expressed (limma analysis *p* < 0.05) in 48 Rb-defective vs. 92 Rb not altered triple-negative breast tumours from the TCGA study [27]. Four highly SCF^SKP^/COP9 complex genes, highlighted in red, demonstrate significantly higher mRNA expression levels in the Rb-defective cell lines. **k** As per **j**, using data from the Metabric study [28]. **l** Box plots illustrating elevated SKP2 or GPS1 (COPS1) mRNA expression in Rb-defective TNBC from both the TCGA [27] and Metabric studies [28]. *p*-values shown calculated with Wilcox rank sum test

When taking all of the highly penetrant Rb synthetic lethal effects identified in the analysis of the *Colt2* data set into account, a significant number of these included genes/proteins known to be associated with Rb function, including the Rb-interacting protein TAF1 (siMEM *p* = 0.016, SLP 92% and SLC 38%, Fig. 4a) [41]. TAF1 encodes the major 250 kDa subunit of the transcription initiation factor, TFIID [42], which binds core promoter regions, including promoter start sites in genes implicated in cell cycle control [43]. We found that over half (*n* = 33) of the highly penetrant Rb synthetic lethal genes encompassed putative TAF1-binding sites, as defined by TAF1 Chip-Seq data [26, 44] a significant enrichment over chance alone (*p* = 2.72 × 10^−13^, Fig. 4b). One mechanistic explanation for these observations might be that many of the highly penetrant Rb synthetic lethal interactions that operate in TNBC are caused by aberrant TAF1 activity, a downstream effect of Rb dysfunction. TAF1 encompasses two protein domains, a kinase and bromodomain, which in principle are pharmacologically tractable. When taken together with the highly penetrant Rb synthetic lethal interaction, this might make TAF1 an attractive target for cancer drug discovery [45–47].

SKP1 and SKP2 were also identified as highly penetrant Rb synthetic lethal effects (Fig. 4c, d, SLP 100% and 92% for SKP1 and SKP2, respectively). Both SKP1 and SKP2 are part of an E3 ubiquitin ligase complex, Skp, Cullin F-box containing complex (SCF^SKP2^), whose activity is greatest during late G_1_/early S phase of the cell cycle. Using ubiquitin ligation, the SCF^SKP2^ complex targets proteins for proteasomal degradation, including the cyclin-dependent kinase inhibitors, p21 and p27 [48]. Three other highly penetrant Rb synthetic lethal effects identified were also associated with the SCF complex (Fig. 4e–g); *COPS1* (aka *GPS1*, SLP 83%, Fig. 4e), *COPS3* (SLP 83%, Fig. 4f) and *COPS4* (SLP 92%, Fig. 4g) encode components of the constitutive photomorphogenesis 9 (COP9) signalosome complex (CSN [49, 50]). CSN regulates the ubiquitin ligase activity of SCF complexes via the deneddylation of the ring finger subunits (e.g., Rbx1) within SCF [51] (Fig. 4i). We also noted that *CKS1B* (CDC28 protein kinase regulatory subunit 1B) also represented a penetrant Rb synthetic lethal partner (SLP 58%, Fig. 4h); together with its co-factor SKP2, CKS1B provides the substrate-specific targeting of p27 by SCF^SKP2^ [52]. When we compared transcriptomic data from RB1-defective TNBCs to those from TNBC with no apparent RB1 defect (Supplementary Data 3 and 4), we noted that RB1-defective TNBCs expressed significantly elevated levels of *SKP2* and *COPS1* (*GPS1*) mRNA (Fig. 4j, k, l, *p* < 0.05 for both TCGA [27] and Metabric [28] data, Wilcox rank sum test), suggesting that in these particular tumours, elevated SKP2 activity might buffer the effects of RB1 dysfunction.

**Fig. 5:**
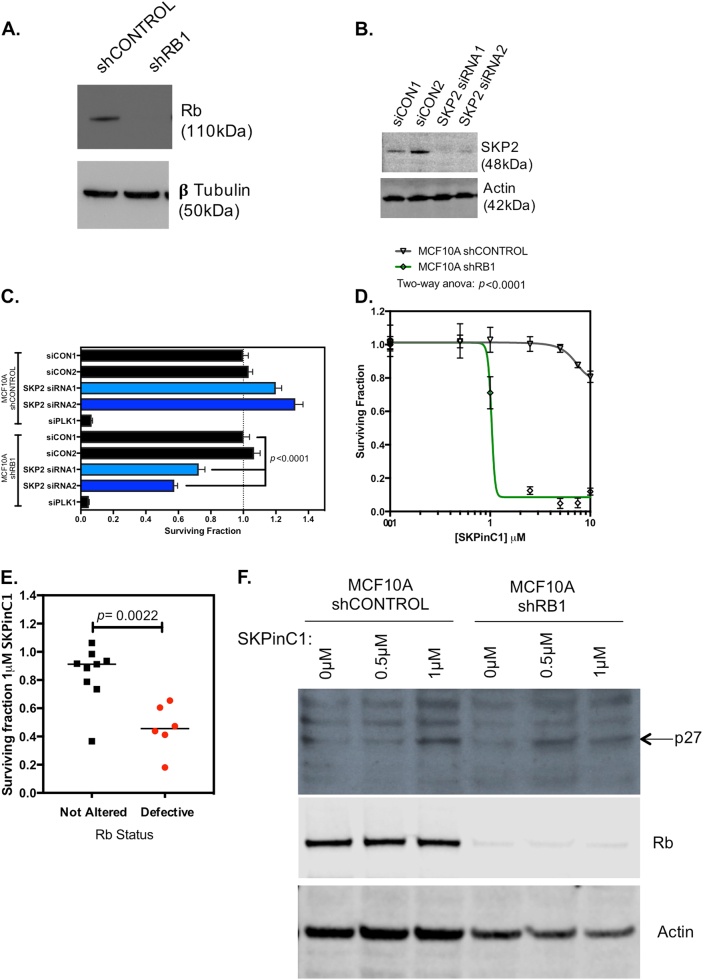
Small-molecule inhibition of SKP2 in Rb-defective breast cell lines is synthetic lethal. **a** Western blot illustrating loss of Rb expression in isogenic MCF10A non-tumour breast epithelial cells with constitutive expression of either a control non-targeting shRNA (shCONTROL) or an Rb-targeting shRNA (shRB1). **b** Western blot illustrating loss of SKP2 protein expression in MCF10A cells 48 h after transfection with SKP2 or control, non-targeting, siRNAs (siCON1 or siCON2). **c** Bar chart illustrating cell inhibitory effects in isogenic MCF10A cells with/without stable expression of Rb shRNA transfected with SKP2 siRNA. Cells were reverse transfected with siRNAs as shown and cultured for 5 continuous days, at which point cell viability was assessed by use of CellTitre-Glo reagent. SKP2 siRNA caused significant cell inhibition (*p* < 0.001, Student’s *t* test) in cells with stable Rb silencing, but not in cells with wild-type Rb expression. **d** Dose response survival curves illustrating the cell inhibitory effects of the SKP2 small-molecule inhibitor, SKPinC1, in isogenic MCF10A cells with/without stable expression of Rb shRNA. Cells were exposed to SKPinC1 for 5 continuous days at which point cell viability was assessed by use of CellTitre-Glo reagent. Rb-defective cells demonstrated a profound sensitivity, compared to Rb wild-type cells (*p* < 0.0001, two-way ANOVA, SF_50_ = 1 and >10 μM in Rb-defective and wild-type cells, respectively). **e** Tumour cell inhibitory effect of SKPinC1 in 13 TNBC TCLs classified according to Rb status. Cells were exposed to 1 μM SKPinC1 as in **h**. Surviving fractions are shown (*p* = 0.0022, Student’s *t* test). **f** Western blot illustrating p27 protein levels in Rb-defective cells exposed to 0.5 and 1 μM SKPinC1 for 24 h

SKP2 directly interacts with Rb [53] and has previously been shown to be required for the hyper-proliferative phenotype of *Rb*-depleted human retinoblastoma cells, via its regulatory control over p27 levels [48]. In cells with normal G_1_/S cell cycle control, Rb binds SKP2, impairing its activity within the SCF^SKP2^ complex; loss of Rb however results in elevated SKP2 activity, a resultant reduction in p27 and p21 protein levels, loss of Cyclin E-CDK2 and Cyclin D-CDK4,6 inhibition and thus progression of cells through the G_1_ restriction point and into S phase [54]. To directly test whether Rb loss in a breast epithelial cell could cause synthetic lethality with SKP2 inhibition, we silenced SKP2 using two different siRNAs in non-tumour breast epithelial MCF10A cells expressing either a shRNA silencing *RB1* or a non-silencing control shRNA (Fig. 5a). Silencing of *SKP2* (Fig. 5b) elicited synthetic lethality in Rb-defective cells but not in Rb-proficient cells (Fig. 5c, Student’s *t* test *p* < 0.0001, Supplementary Figure 2). We also found that the toolbox SKP2 inhibitor SKPinC1, which impairs the binding of phosphorylated p27 to the substrate recognition pocket formed by SKP2 and CKS1B [55], had a profound synthetic lethal effect on Rb-defective MCF10A cells, but minimal effects in Rb wild-type cells (*p* < 0.001 two-way analysis of variance (ANOVA), Fig. 5d) and induced apoptosis in Rb-defective cells (Supplementary Figure 3). We also assessed SKPinC1 sensitivity in 13 TNBC TCLs, and found that Rb-defective TCLs were more sensitive than TCLs with no apparent Rb defect (Fig. 5e and *p* < 0.0022, Student’s *t* test). In addition, we confirmed that exposure of MCF10A cells with SKPinC1 inhibitor led to a stabilisation of p27 protein levels (Fig. 5f). Targeting of p27 by siRNA also partially reversed the inhibitory effect of SKPinC1 (Supplementary Figure 4), suggesting that the effect of SKPinC1 was p27-dependent. Taken together, these observations suggested that SKP2 small-molecule inhibition, could in principle, elicits the highly penetrant Rb synthetic lethal effect seen in TNBC tumour cells with RNA interference reagents.

We also assessed whether other highly penetrant synthetic lethal effects operated in the RB1 isogenic MCF10A system. Using an arrayed siRNA screen we tested all 54 genes that we identified as highly penetrant (>80% penetrance) RB-synthetic lethal effects in TNBC cell lines in the Colt2 study along with an additional four controls (e.g., E2F3) for a total of 58 genes. We found that over half of the identified dependencies (55%) were observed in the isogenic system (Supplementary Figure 5), including profound synthetic lethal effects associated with *TIMELESS*, *PCDH1*, *PITRM1*, *E2F3*, *SMN2* and *TCOF1*. This suggests that these effects can be specifically associated with RB loss, and that they are not an artefact of either the shRNA library used in Colt2 or the pooled screening approach. It seemed possible that differences in the proliferative rate of RB1-defective vs. wild-type MCF10A cells could account for the synthetic lethal effects observed. However, we found that the proliferative rate of RB1-defective and wild-type MCF10A cells was similar (Supplementary Figure 6), suggesting this might not have a significant bearing on the synthetic lethal effects identified. Moreover, analysis of previously published doubling times for 17 TNBC cell lines identified no significant difference between RB1-defective and RB1-proficient models (Mann-Whitney *U*-test *p* = 0.4) [56].

**Fig. 6:**
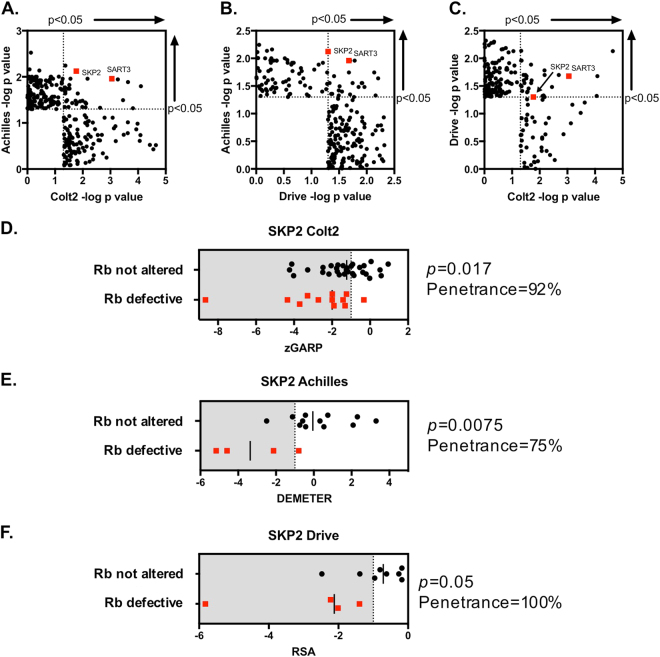
SKP2 identified as a highly penetrant Rb synthetic lethal effect in multiple, independently derived, data sets. **a**–**c** Scatter plots comparing *p*-values (−log_10_) for Rb synthetic lethal effects identified in Colt2 [22], Achilles [23] and DRIVE [29] data sets. *p* < 0.05 effects in any single screen are shown. *p* < 0.05 effects in two screens are shown in the top right hand quadrant of each plot. SKP2 and SART3, which were identified in all three screens as synthetically lethal with Rb defects, are highlighted in red. *p* < 0.05 effects in Colt2 data were identified by siMEM, followed by *Z* score and penetrance filtering (Fig. 2a); *p* < 0.05 effects in DRIVE and Achilles data were identified by median permutation *t* test, followed by *Z* score and penetrance filtering (Fig. 2a). **d** Scatter plots illustrating *Z* scores in 42 TNBC TCLs for SKP2 from the Colt2 data analysis. **e** Scatter plots illustrating *Z* scores in 16 TNBC TCLs for SKP2 from the Achilles data analysis. **f** Scatter plots illustrating *Z* scores in 12 TNBC TCLs for SKP2 from the Drive data analysis

We also assessed which of the highly penetrant Rb synthetic lethal effects identified in our analysis of the *Colt2* data were also identified as highly penetrant effects in the TNBC TCLs in two other shRNA screens: Achilles [23] and DRIVE [29]. Comparing the *p* < 0.05 Rb penetrant synthetic lethal effects between the three data sets, we noted that SKP2 was one of two synthetic lethal effects identified in all three screens, the other effect being associated with SART3, the RNA splicing factor (Fig. 6a-c, Supplementary Data 9 and 10, 11). In each screen, SKP2/Rb synthetic lethality was highly penetrant with SLP scores of 92% (Colt2), 75% (Achilles) and 100% (DRIVE data set, Fig. 6d–f). Whilst the TNBC TCLs described in these three data sets are partially overlapping, the shRNA libraries used were independently designed and synthesised, and the screens independently carried out. As such, identifying SKP2 as a highly penetrant Rb synthetic lethal effect in all three data sets suggested that this effect was somewhat independent of the shRNA reagents used.

### Rb/SKP2 synthetic lethality operates in tumour cells from lung and other cancer histologies

**Fig. 7:**
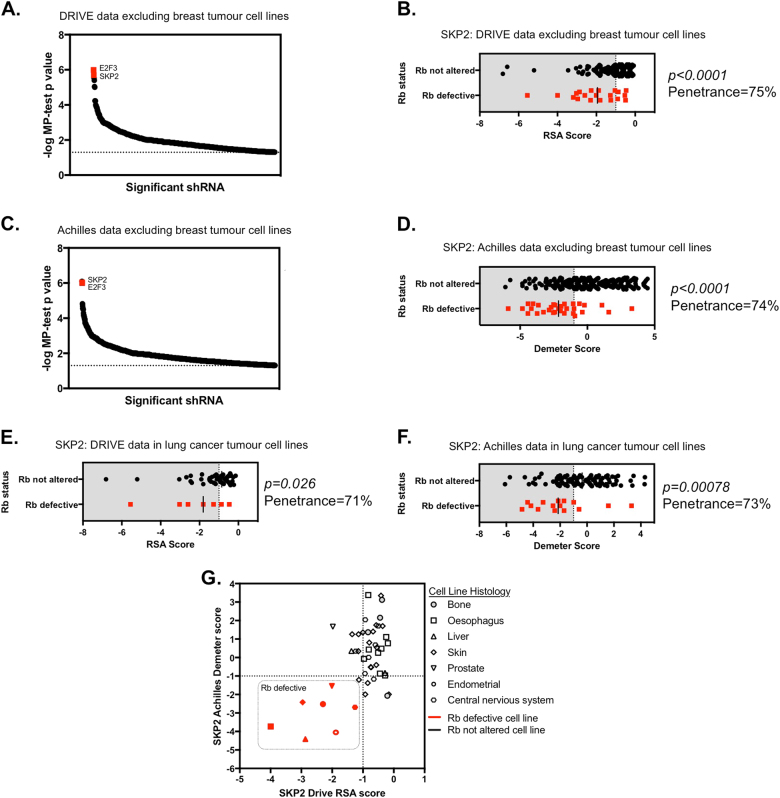
SKP2 identified as a highly penetrant Rb synthetic lethal effect in other histologies in two independently derived data sets. **a** Scatter plot of 775 *p* < 0.05 significant Rb synthetic lethal effects identified from the MP test analysis of 373 non-breast cancer TCLs in the Drive study (step one in Fig. 1a). All 775 *p* < 0.05 effects are ranked ordered by MP test *p* value. SKP2 and E2F3 are highlighted. **b** Scatter plot illustrating RSA scores in 373 non-breast TCLs with Rb annotation for SKP2 sensitivity from the Drive data analysis. **c** Scatter plot of 1467 *p* < 0.05 significant Rb synthetic lethal effects identified from the MP test analysis of 467 non-breast TCLs in the Achilles study (step one in Fig. 1a). All 1467 *p* < 0.05 effects are ranked ordered by MP test *p* value. SKP2 and E2F3 are highlighted. **d** Scatter plots illustrating Demeter scores in 1467 non-breast TCLs with Rb annotation for SKP2 sensitivity from the Achilles data analysis. **e**, **f** Scatter plots illustrating RSA and Demeter scores in 63 and 115 lung TCLs with Rb annotation for SKP2 sensitivity from the Drive and Achilles studies, respectively. **g** Scatter plot of intersect of cell line between the two data sets showing SKP2 sensitivity in Drive RSA scores (*x* axis) and Achilles Demeter scores (*y* axis) for selected histologies with only a single Rb-defective line. This graph illustrates a trend between Rb defects and sensitivity to SKP2 shRNA across seven different histotypes

Rb defects are relatively prevalent in TNBC, but are not unique to this cancer subtype and are also evident in in many tumour types, including, for example, small-cell lung cancers, bladder carcinomas, osteosarcomas, glioblastomas, endometrial carcinomas and retinoblastomas [7, 8, 57–59]. To estimate whether the highly penetrant Rb/SKP2 synthetic lethality in TNBC extended to other cancer histologies, we re-analysed the results of two recent large-scale shRNA screens that encompass TCLs derived from non-breast cancer histologies. Project DRIVE includes 373 non-breast TCLs with available Rb mutation and copy number status that were derived from 18 different cancer histologies [29]. In an analysis of the DRIVE data set that excluded the breast TCLs, we found a significant association between *RB1* mutation/deletion and sensitivity to *SKP2* shRNA (Fig. 7a, b; median permutation (MP)-test *p* < 0.0001, SLP = 75%). Of over 6000 genes tested, only *E2F3* (MP test *p* < 0.0001) showed a stronger association with Rb status (Fig. 7a). Similarly, project Achilles includes 467 non-breast TCLs with available Rb mutation and copy number status, derived from 20 different cancer histologies [23]. In an analysis of the Achilles data set that excluded the breast TCLs, we also found a significant association between *RB1* mutation/deletion and sensitivity to *SKP2* shRNA (Fig. 7c, d; MP test *p* < 0.0001, SLP = 74%). Of over 17 000 genes, SKP2 synthetic lethality was the second most significant effect associated with Rb loss, after *CDK2* (MP test *p* < 0.0001) with *E2F3* being the third most significant effect (Fig. 7c). The elevated penetrance of the Rb/SKP2 synthetic lethal effect in tumour cell models other than those derived from breast cancer suggested that this genetic dependency might operate in multiple histologies. When we considered the specific cancer histology of TCLs, rather than analysing these *en mass*, we found in both the Achilles and DRIVE data sets the Rb/SKP2 synthetic lethality was detectable in TCLs derived from lung cancers (*p* = 0.026/SLP = 71% and *p* = 0.0008/SLP = 73% for DRIVE and Achilles, respectively; Fig. 7e, f). However, we do note that the relatively small number of Rb-defective TCLs from non-lung cancer histologies in these data sets might impair the ability to detect the Rb/SKP2 synthetic lethality. For example, in both DRIVE and Achilles data sets, we noted that Rb-defective TCLs derived from prostate cancer, osteosarcoma, liver cancer and oesophageal cancer, exhibited sensitivity to SKP2 shRNA (Fig. 7g).

## Discussion

There is now a relatively long-standing history of using genetic concepts such as synthetic lethality to identify novel therapeutic targets for the treatment of cancer [60]. In part these efforts have been successful, with synthetic lethal treatments [16] and drugs that exploit oncogene addiction effects now being approved for the treatment of the clinical disease [61]. However, despite these advances, one challenge to this approach has been in identifying highly penetrant synthetic lethal effects that associate with the presence of a molecular biomarker. Here we describe efforts to identify highly penetrant synthetic lethal effects associated with loss of the tumour suppressor *Rb* in TNBC. Following the classification of 42 TNBC TCLs according to Rb status, we interrogated genome-scale and focussed gene set shRNA screening data sets, identified candidate synthetic lethal effects and then used stringent filters to triage from the list of candidate synthetic lethal effects those most likely to represent highly penetrant effects. This approach allowed us to identify a number of highly penetrant synthetic lethal effects, many of which are associated with known functions of Rb and/or associated with Rb-interacting proteins. These included TAF1 and TAF1 target genes as well as members of the SCF^SKP2^ complex.

We note that there are considerable caveats associated with the approach we have taken to identifying highly penetrant Rb-related synthetic lethal effects in TNBC and the interpretation of the data; understanding these caveats is critical to the use of the information we provide in this work. Primary amongst these is that we have exclusively used data from in vitro genetic screens to identify synthetic lethal effects and assess their penetrance. It seems likely that some of the effects identified in our analysis only operate in the context of two-dimensional in vitro culture and are abrogated, and therefore appear less penetrant, in three-dimensional and/or in vivo settings. Such a possibility thus provides the rationale for also assessing synthetic lethal effects, and assessing their penetrance, in in vivo models of cancer. Second, there is little way of effectively estimating the true false negative rate of our approach; it is entirely possible that we have not identified real, highly penetrant, Rb-related synthetic lethal effects either because the RNA interference reagents used in genetic screens ineffectively inactive the genes they are designed to target or because some other form of gene/protein inactivation (e.g., catalytic inhibition of the target protein rather than gene silencing) is required to elicit a synthetic lethal effect. Nevertheless, the identification in multiple, independently conducted, genetic screens of the highly penetrant Rb/SKP2 synthetic lethal effect, and its recapitulation with a small-molecule inhibitor, suggests that this highly penetrant synthetic lethality effect is unlikely to be a false positive.

As well as targeting Rb-defective TNBC, the potential for using SKP2 inhibition in other cancer histologies associated with Rb defects is considerable. For example, the hyper-proliferative phenotype of *Rb*-depleted human retinoblastoma cells and mouse melanotrophs is dependent upon the SKP2 [48, 62] and the SKP2/CKS1 pocket inhibitor, SKPinC1, inhibits *Rb/p53*-defective mouse prostate tumour cell organoids [63]. Finally, large-scale shRNA screens in TCLs from a variety of cancer histologies (and our analysis described in Fig. 6) suggest that the dependency of Rb-defective TCLs upon SKP2 might extend beyond models of TNBC. Whether the high penetrance of the Rb/SKP2 synthetic lethality seen in models of TNBC extends to each of these histologies remains to be seen, but our initial analysis in Fig. 6 suggests that highly penetrant effects might also be apparent in lung cancer.

It might be interesting to speculate what characteristics might differentiate a highly penetrant synthetic lethal effect that operates in cancer from less-penetrant effects; being able to understand the factors that distinguish one from the other might eventually allow highly penetrant synthetic lethal effects in cancer to be predicted from first principles, thus reducing the reliance upon large-scale genetic screens in multiple TCLs to empirically establish penetrance. From our relatively unbiased analysis of genome-scale shRNA screens, it is perhaps striking that many of the highly penetrant Rb synthetic lethal effects we identified (e.g., SKP1, SKP2, CKS1B, COPS1, COPS2 and COPS3) have two characteristics: (i) they are closely, rather than distally, involved in controlling an essential process in highly proliferating tumour cells, namely G_1_ cell cycle progression by Cyclin/CDK activity; and (ii) this process is also closely, rather than distally, controlled by the synthetic lethal partner, Rb. It seems reasonable to think that synthetic lethal interactions that control essential processes in cancer cells via small-world networks (i.e., those than contain a relatively small number of nodes between synthetic lethal partners and proteins involved in essential processes) might be less likely to be reversed, and therefore more likely highly penetrant, than synthetic lethal effects that control essential processes via distal molecular mechanisms that involve larger molecular networks with multiple intervening nodes. Whether this turns out to be a general principle or not remains to be seen but already others have started to establish that many synthetic lethal effects associated with tumour suppressor genes other than Rb can be classified into a defined, and relatively small number of classes, including those between paralogs and those between genes in the same molecular pathway [23]. This suggests that some of the principles that govern how synthetic lethal effects operate in tumour cells can indeed be established.

## Materials and methods

### Cell lines, compounds and siRNA

All cell lines were obtained from the American Type Culture Collection and maintained according to the supplier’s instructions. Cell lines were routinely STR typed and mycoplasma tested. Cell lines were grown and transfected with individual and SMARTpool siGENOME siRNA (Dharmacon) and transfected using RNAiMAX (Invitrogen) reagent as described in ref. [21]. Transfection efficiency was monitored using positive control (siPLK1) and two different negative control siRNAs (siCON1 and siCON2; Dharmacon, catalogue numbers D-001210-01-20 and D-001206-14-20). SKPinC1 inhibitor was purchased from Tocris (no. 4817) and was solubilised as a 10 mM stock solution in dimethyl sulfoxide (DMSO).

### Western blot analysis

Whole-cell protein lysates were extracted from cells by lysis with NP250 buffer (20 mM Tris (pH 7.6), 1 mM EDTA, 0.5% NP40 and 235 mM NaCl). In gene silencing experiments, cells were cultured for 48 h after siRNA transfection, at which point lysates were generated. Following a 20 min incubation at 4 °C, lysates were centrifuged and supernatents collected. Electrophoresis was performed using Novex precast Bis-Tris gels (Invitrogen) and gels were blotted onto nitrocellulose filters as described previously [64]. Blots were immunoblotted in 5% (w/v) milk at 4 °C overnight using the following primary antibodies: anti-Rb1 (1/1000 (v/v) dilution in 5% (v/v) milk, New England Biolab, 9309); anti-p16 (1/1000, abcam); anti-SKP2 (1/1000, New England Biolab, 4358); anti-p27 (1/1000, New Engand Biolab, 2552); anti-tubulin (1/1000, abcam); and anti-actin (1/1000, Santa Cruz, sc-1616). After washing, blots were incubated 1 h at room temperature with secondary antibodies (Li-COR) diluted 1/10 000 (v/v) in 5% (w/v) milk. Protein bands were visualised and quantified using the Odyssey FC imaging system and ImageStudio software (Li-COR).

### Tumour and cell line expression analysis

Limma [65] was performed to identify differentially expressed genes RB1-defective vs. RB1 not altered TCLs, using data from refs. [22, 25]. A design matrix with the cell line RB1 classifications was created and a linear model was fitted to expression values of each gene. Next, an empirical Bayes method was used to obtain more precise gene-wise variability estimates between the two groups. Differential expression between groups was represented as log fold change scores with associated *p*-values and adjusted *p*-values. Publically available cell line mRNA expression data sets used in this study include Marcotte [22] and CCLE [25], as indicated.

For the analysis of TNBC tumours, mRNA expression, DNA copy number and somatic mutation profiles for TCGA tumours were downloaded from GDAC (https://gdac.broadinstitute.org/), release 2016_01_28. GISTIC v2 level 4 data were used for copy number analysis and log_2_ ratios were converted to genomic gains/amplifications, neutral and loss/deletion states using threshold of ±log_2_(3/2). Raw Metabric data files were downloaded from the European Genome-phenome Archive (EGA; study ID EGAS00000000083). The Metabric breast cancer data set was pre-processed, summarised and quantile-normalised from the raw expression files generated by Illumina BeadStudio (R packages: beadarray v2.4.2 and illuminaHuman v3.db_1.12.2). Probe to gene-level mapping was performed by keeping the most variable (standard deviation) probe. Metabric copy number calls were used as published in the original study [66].

For TCGA breast cancer cohort, previously published [67] TNBC6 and TNBC4 calls were used resulting in 140 patients with matched mRNA, copy number and mutation profiles. For the Metabric cohort, TNBC6 calls were successfully derived from TNBC subtyping portal [68] (http://cbc.mc.vanderbilt.edu/tnbc) for 187 patients with matched mRNA and copy number profiles. In the TCGA cohort, TNBC samples with RB1 mRNA *Z* score < −1 were regarded as Rb-low, RB1 copy number log_2_ ratio < −0.585 were regarded as Rb loss and samples with RB1 truncating mutations were considered as Rb inactivated. This resulted in 48 Rb-defective and 92 Rb not altered TNBC samples. In the Metabric cohort, samples with RB1 mRNA *Z* score < −1 were regarded as Rb-low, whilst samples with RB1 loss/deletions were regarded as Rb loss. This resulted in 55 Rb-defective and 132 Rb not altered TNBC samples.

Differential gene expression analysis on TCGA TNBC samples was performed using limma voom [65].

### Association testing

The siMEM was used to measure the essentiality of genes in the Colt2 genome-wide shRNA dropout screen [22]. siMEM uses hierarchical linear regression and considers level of each shRNA to be a regression function of its initial abundance over time, baseline trend in abundance over time and difference in abundance trend between samples sharing a common feature. siMEM results are represented as the difference between the dropout rate of hairpins in the two cell line groups being compared.

For analysis of the DRIVE and Achilles shRNA screen data sets, where only a single screen time point was collected, a one-sided MP test was used to identify associations between *RB1* status of cell lines and their sensitivities to shRNA targeting of genes. For each gene, the observed difference between the median of scores of RB1-defective and RB1 not altered groups of TCLs was compared. A total of one million random samples with the same sample size as in the two groups were created. The differences in the medians of the groups were calculated for each random sample and the statistical significance of the difference was determined.

### Cell viability assays

In all, 500 cells per well were seeded into 384-well plates. After 24 h cells were exposed to increasing concentrations of SKPinC1 inhibitor diluted in DMSO using an Echo 550 liquid handler (Labcyte). Cells were incubated with the inhibitor for 5 days after which cell viability was estimated with CellTitre-Glo reagent (Promega). For gene silencing experiments cells were incubated for 5 days following siRNA transfection prior to viability assessment. Luminescence readings from drug-exposed cells were normalised to those from DMSO-exposed cells to calculate surviving fractions (SFs). SFs were used to derive four-parameter logistic regression dose/response curves using Graphpad Prism software, as per the manufacturer’s instructions. Apoptosis was measure using Caspase-Glo 3/7 reagent (Promega).

### Statistical analysis

Unless otherwise stated all data are represented here as mean ± standard error using Graphpad Prism Software (La Jolla, CA). All conditions were performed in triplicate in at least triplicate experiments. Statistical significance was measured using either Student’s *t* test, Pearson coefficient correlation, Mann-Whitney *U*-test or two-way ANOVA. *p* < 0.05 was considered significant.

## Electronic supplementary material

Supplementary Figure Legends

Supplementary Figure 1

Supplementary Figure 2 and 3

Supplementary Figure 4

Supplementary Figure 5

Supplementary Figure 6

Supplementary Data Table 1

Supplementary Data Table 2

Supplementary Data Table 3

Supplementary Data Table 4

Supplementary Data Table 5

Supplementary Data Table 6

Supplementary Data Table 7

Supplementary Data Table 8

Supplementary Data Table 9

Supplementary Data Table 10

Supplementary Data Table 11

Supplementary Data Table 12

Supplementary Data Table 13
