# Supplementary material for: Identification of highly penetrant Rb-related synthetic lethal interactions in triple negative breast cancer
Source: Oncogene. 2018 Jun 18;37(43):5701–18. doi: 10.1038/s41388-018-0368-z (PMC6202330; doi:10.1038/s41388-018-0368-z)
Supplement: Supplementary file 1 — Supplementary Figure Legends [file 41388_2018_368_MOESM1_ESM.docx]

**Supplemental Information**

**Supplementary Figure 1.** GO biological processes identified from the list of highly penetrant Rb SL genes (>80% penetrant).

**Supplementary Figure 2.** Bar chart illustrating cell inhibitory effects in isogenic MCF10A shCONTROL and MCF10A shRB1 cell lines transfected with two SKP2 siRNA (siRNA 1 and siRNA 2). Cells were reverse transfected with siRNAs as shown and cultured for five continuous days, at which point cell viability was assessed by use of CellTitre Glo reagent. SKP2 siRNA caused significant cell inhibition (p<0.001, Student’s t test) in cells with stable Rb silencing, but not in cells with wild type Rb expression.

**Supplementary Figure 3.** SKPinC1 inhibitor induces apoptosis in RB1 deficient MCF10A cells. MCF10A shCONTROL and shRB1 cells were exposed to either DMSO (the drug vehicle) or 1μM SKPinC1 inhibitor for 48 hours. After this time, apoptosis was estimated using Caspase-Glo 3/7assay reagent. The bar graph shows relative Caspase 3/7 levels compared to each cell lines DMSO control. Error bars represent SEM from three independent experiments. p value calculated using Student’s t test.

**Supplementary Figure 4.** p27 is required for sensitivity to SKPinC1 inhibitor in RB1 defective cells. MCF10A shRB1 cells were transfected with siCON1 or siRNA targeting p27. 48 hours after transfection, cells were exposed to either DMSO or 5 μM of SKPinC1 for four days. Cell viability was then assessed using Cell-Titre Glo reagent and surviving fractions were calculated. Silencing of p27 partially reversed the synthetic lethality elicited by SKPinC1.

**Supplementary Figure 5.** Revalidation of highly penetrant *RB1* synthetic lethal effects in a *RB1* isogenic cell line model. **A.** MCF10A shRB1 cells (a clone with stable gene silencing of *RB1*) or MCF10A shCONTROL cells (a clone with stable expression of a non-targeting shRNA) were transfected with siRNA designed to target one of 58 candidate RB1 synthetic lethal genes. Five days following transfection cell viability was assessed using CellTitre-glo reagent. Surviving fractions were calculated to the median of the negative control siRNA for each cell line and the median difference (median surviving fraction of siRNA for gene “X” in MCF10A shCONTROL cells - median surviving fraction of siRNA for gene “X” in MCF10A shRB1 cells) and a *p* value (Student’s t test) were calculated. The scatter plot shows the median difference in surviving fraction (on the x axis), where a negative value indicates selective inhibition of the shRB1 cell model and –log10(p value) on the y axis. 32 out of the 58 siRNA (55%) demonstrated a *p*<0.05 RB1 synthetic lethal effect (i.e. selectively targeting the shRB1 cells). **B-G.**  Dot plots illustrating surviving fractions of the six most *RB1*-selective effects; *TIMELESS, PCDH1, PITRM1, E2F3, SMN2* and *TCOF1***.**

**Supplementary Figure 6. Growth kinetics of MCF10A RB1 silenced cells compared to RB1 expressing MCF10A cells. A.** MCF10A cells were seeded into 24-well plates and their growth was monitored every 12 hours using an Incucyte platform. The graph shows the relative confluency of each cell line during a seven-day period. **B.** Analysis of published^25^ doubling times for 17 TNBC cell lines. No significant differences in doubling times are seen between RB1 defective and RB1 proficient models (Mann-Whitney U-test *p*=0.4)
