# Supplementary figures and images for: Identification of highly penetrant Rb-related synthetic lethal interactions in triple negative breast cancer
Source: Oncogene. 2018 Jun 18;37(43):5701–18. doi: 10.1038/s41388-018-0368-z (PMC6202330; doi:10.1038/s41388-018-0368-z)

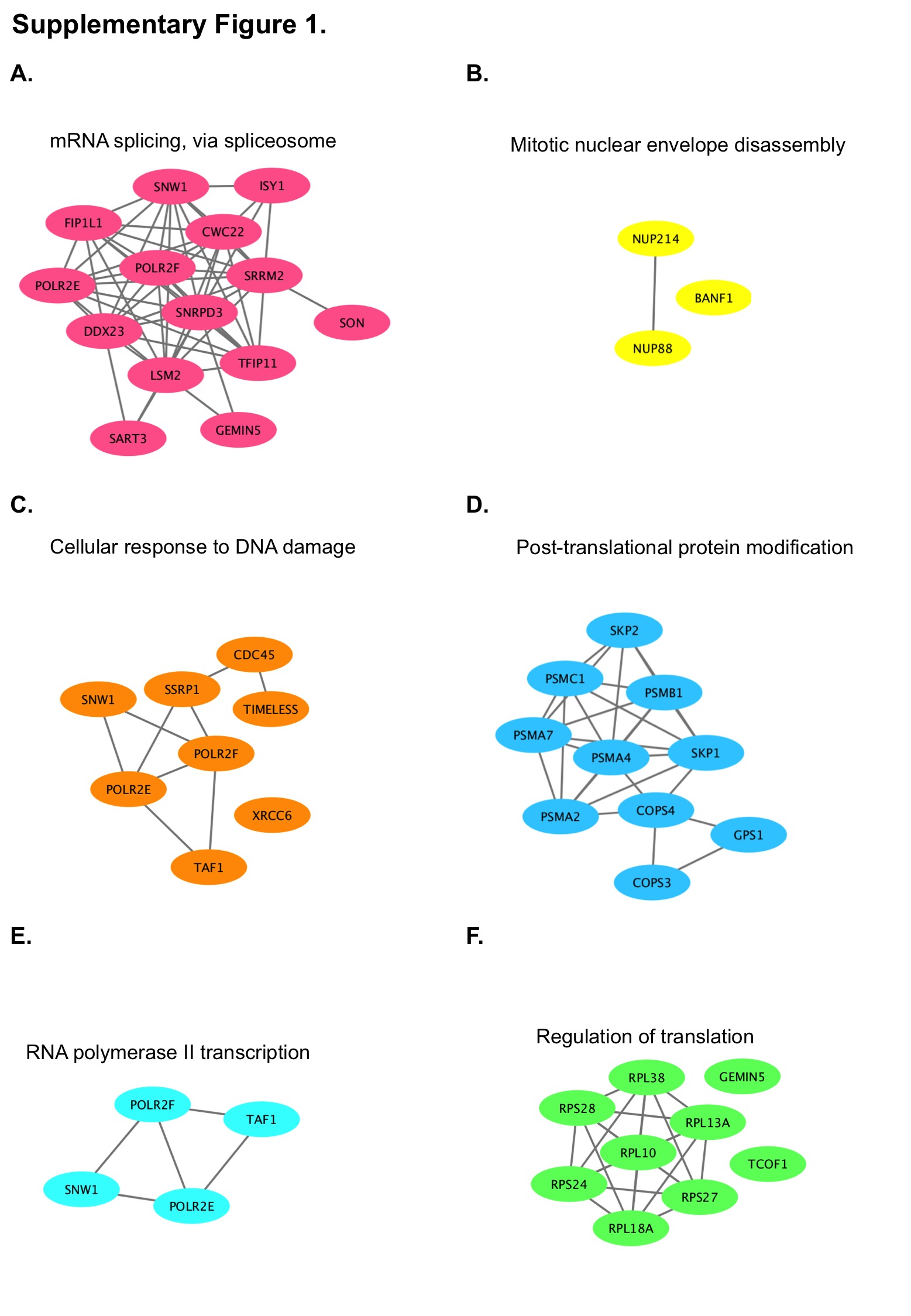

Supplement: Supplementary file 2 — Supplementary Figure 1 [file 41388_2018_368_MOESM2_ESM.jpg]

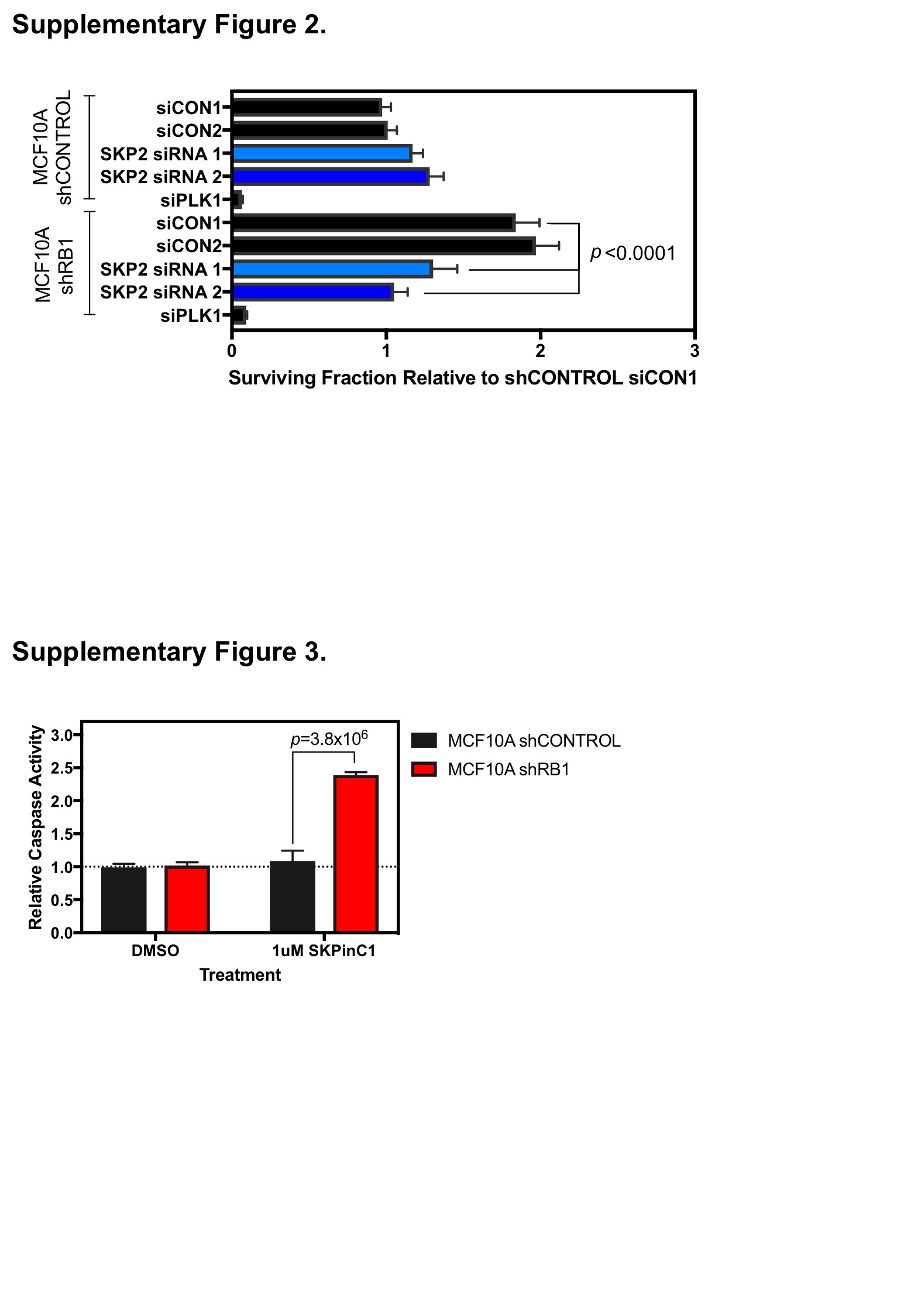

Supplement: Supplementary file 3 — Supplementary Figure 2 and 3 [file 41388_2018_368_MOESM3_ESM.jpg]

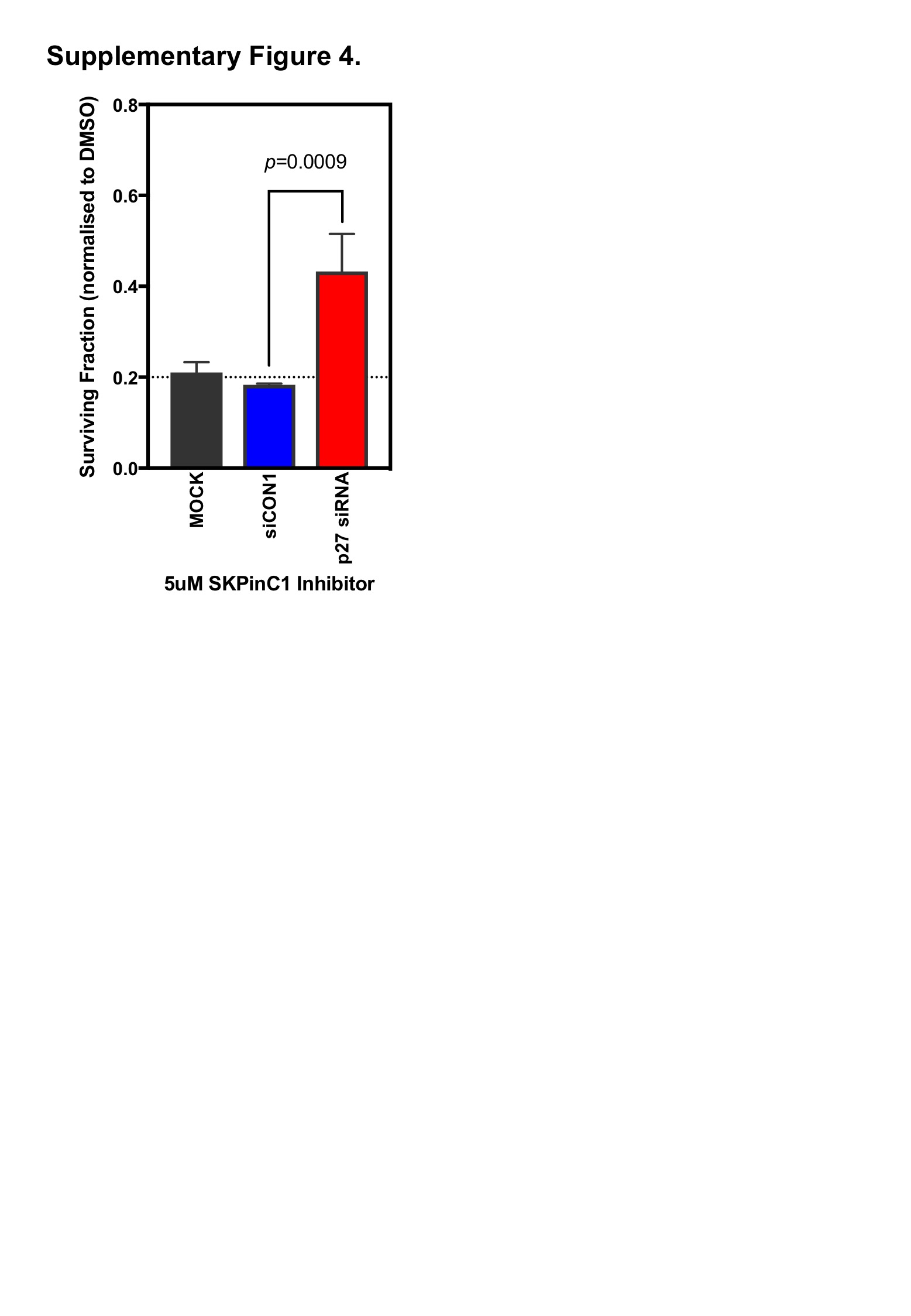

Supplement: Supplementary file 4 — Supplementary Figure 4 [file 41388_2018_368_MOESM4_ESM.jpg]

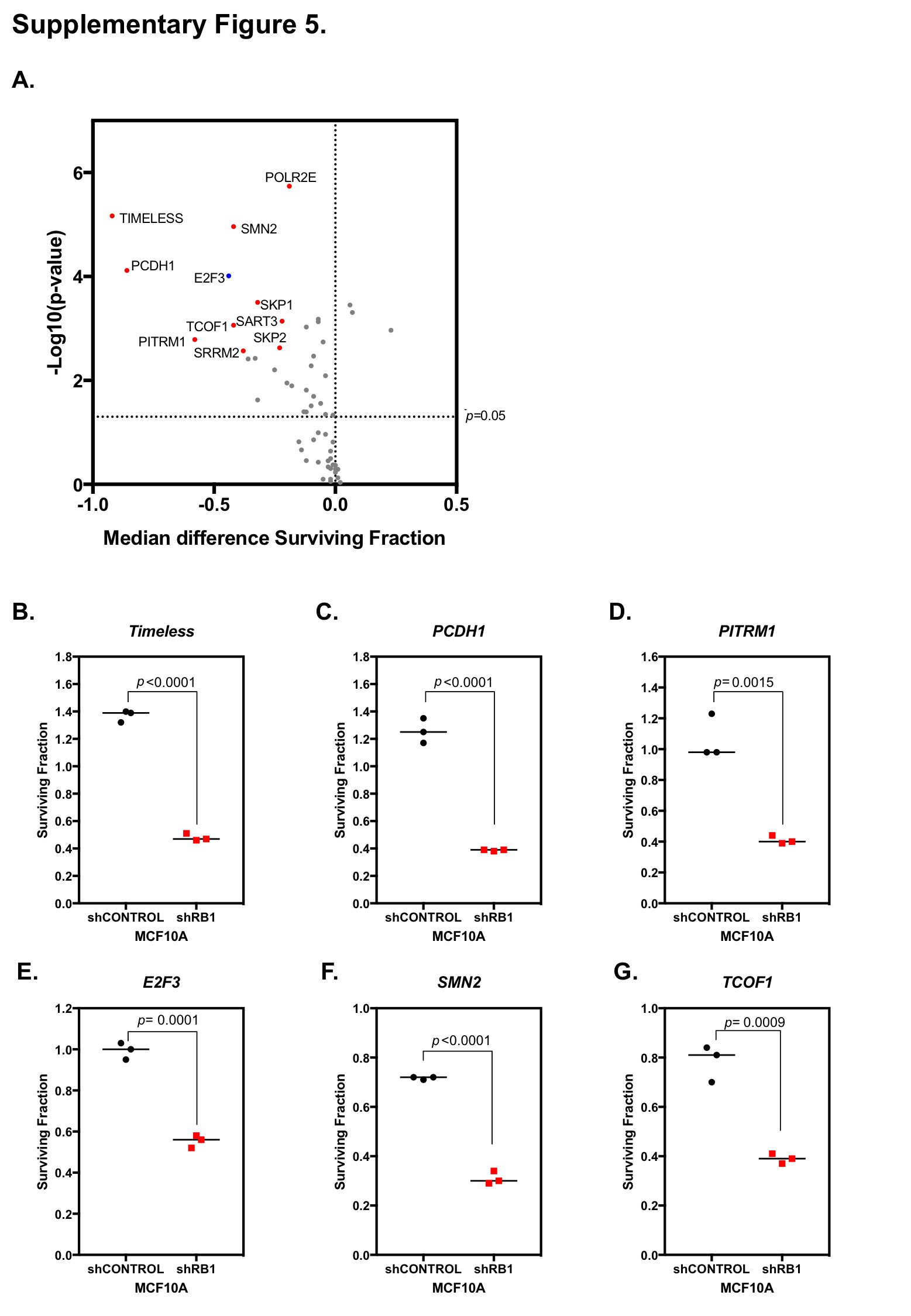

Supplement: Supplementary file 5 — Supplementary Figure 5 [file 41388_2018_368_MOESM5_ESM.jpg]

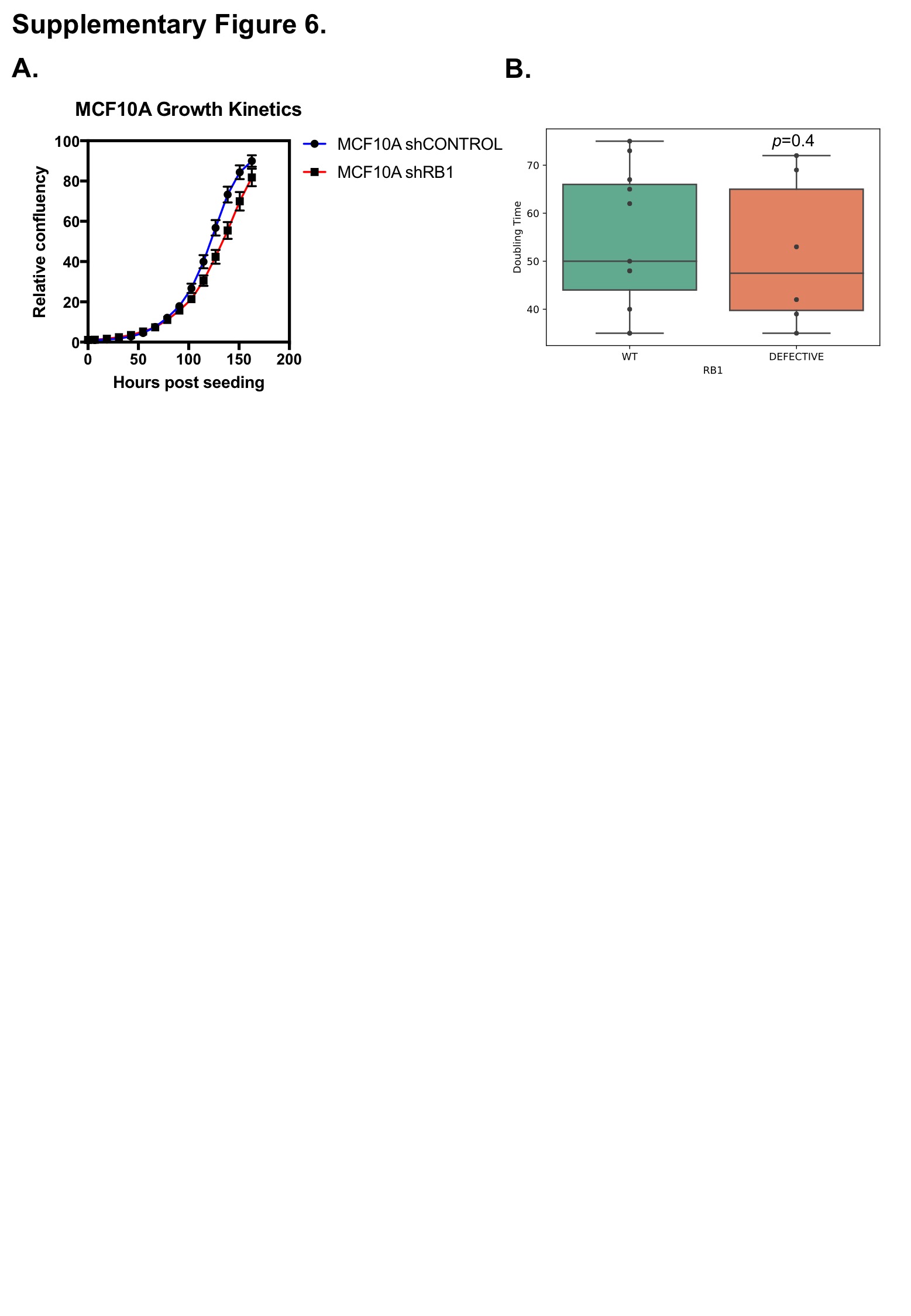

Supplement: Supplementary file 6 — Supplementary Figure 6 [file 41388_2018_368_MOESM6_ESM.jpg]
